# Non-Invasive Diagnosis of Malignancies Based on the Analysis of Markers in Exhaled Air

**DOI:** 10.3390/diagnostics10110934

**Published:** 2020-11-11

**Authors:** Vladimir I. Chernov, Evgeniy L. Choynzonov, Denis E. Kulbakin, Ekaterina N. Menkova, Elena V. Obkhodskaya, Artem V. Obkhodskiy, Aleksandr S. Popov, Evgeniy O. Rodionov, Victor I. Sachkov, Anna S. Sachkova

**Affiliations:** 1 Tomsk National Research Medical Center of the Russian Academy of Sciences, Cancer Research Institute, 5 Kooperativny Street, 634009 Tomsk, Russia; chernov@tnimc.ru (V.I.C.); choynzonov@tnimc.ru (E.L.C.); kulbakin_d@mail.ru (D.E.K.); katushamenkova123@gmail.com (E.N.M.); rodionov_eo@oncology.tomsk.ru (E.O.R.); 2 Laboratory of Chemical Technologies, National Research Tomsk State University, 36 Lenin Avenue, 634050 Tomsk, Russia; lenaobx@yandex.ru (E.V.O.); art707@tpu.ru (A.V.O.); asptomsktpu@gmail.com (A.S.P.); 3 School of Nuclear Science & Engineering, National Research Tomsk Polytechnic University, 30 Lenin Avenue, 634050 Tomsk, Russia; asachkova@tpu.ru

**Keywords:** malignancy, cancer, markers, non-invasive diagnosis, exhaled air, sensor-based gas analyzer, neural network

## Abstract

Novel non-invasive methods for the diagnosis of malignancies should be effective for early diagnosis, reproducible, inexpensive, and independent from the human factor. Our aim was to establish the applicability of the non-invasive method, based on the analysis of air exhaled by patients who are at different stages of oropharyngeal, larynx and lung cancer. The diagnostic device includes semiconductor sensors capable of measuring the concentrations of gas components in exhaled air, with the high sensitivity of 1 ppm. The neural network uses signals from these sensors to perform classification and identify cancer patients. Prior to the diagnostic procedure of the non-invasive method, we clarified the extent and stage of the tumor according to current international standards and recommendations for the diagnosis of malignancies. The statistical dataset for neural network training and method validation included samples from 121 patients with the most common tumor localizations (lungs, oropharyngeal region and larynx). The largest number of cases (21 patients) were lung cancer, while the number of patients with oropharyngeal or laryngeal cancer varied from 1 to 9, depending on tumor localization (oropharyngeal, tongue, oral cavity, larynx and mucosa of the lower jaw). In the case of lung cancer, the parameters of the diagnostic device are determined as follows: sensitivity—95.24%, specificity—76.19%. For oropharyngeal cancer and laryngeal cancer, these parameters were 67.74% and 87.1%, respectively. This non-invasive method could lead to relevant medicinal findings and provide an opportunity for clinical utility and patient benefit upon early diagnosis of malignancies.

## 1. Introduction

Cancer is the second most prevalent cause of mortality in the population of Russia (16.1%; 2017—15.9%), right after circulatory diseases (46.8%; 2017—47.3%), and followed by injuries and poisoning (7.9%; 2017—8.4%) [1]. Lung, laryngeal and oropharyngeal cancer are the most common nosologies in the structure of cancer incidence in the Russian Federation, and are associated with high disability and lethality rates. For example, the increase in the incidence among the male population in Russia was 36.35% for oral cancer, 29.97% for pharyngeal cancer, and 2.86% for laryngeal cancer. For the female population of Russia, the corresponding trends in incidence for the period from 2008 to 2018 were 56.23% for oral cancer, 43.2% for pharyngeal cancer, and 24.74% for laryngeal cancer.

In 2018, more than two million new cases and 1.7 million deaths from lung cancer were reported, representing 14% of the total number of new cancer cases according to global estimates, and 20% of all cancer deaths [2]. The prognosis for lung cancer remains poor; even with sufficient resources, the five-year survival ranged from 32.9% in Japan down to 13.3% in the UK during the period 2010–2014 [3]. Compared to other common cancers, such as prostate and breast cancer, lung cancer has much lower survival rates, and despite advances in radiological imaging [4].

Such disappointing morbidity and mortality indicators are associated with the fact that in most patients (up to 50–60%) cancer is detected at an advanced stage (T3–T4). This is explained by patients being late in seeking medical attention on the one hand, and by the limitations of existing methods of early cancer diagnosis on the other. For example, most early diagnosis methods are invasive, operator-dependent and use expensive procedures. Therefore, in some cases, such methods are inaccessible to a wide population and do not meet the principles of up-to-date cancer screening [5].

Today, the recommended examination strategy for patients with head and neck tumors is based on the use of endoscopic, radiological and morphological diagnostic methods [6]. The effectiveness of these diagnostic methods depends both on the availability of instrumental and laboratory diagnostics methods, and on the experience of diagnosticians. The recommended diagnostic methods are aimed at the timely detection of tumor processes in the early stages, and effective treatment, in order to achieve good clinical results, which, due to the above-mentioned shortcomings, are not readily achievable. In the treatment of patients with locally advanced cancers of the oropharyngeal region and larynx, a whole complex of negative consequences is often presented, such as disability, physiological dysfunction, severe cosmetic damage, and the development of psychoemotional trauma [7].

The above-mentioned reasons and requirements for modern screening methods for diagnosing oropharyngeal, larynx and lung cancers motivate clinical oncologists and specialists in related fields to find effective methods for the early diagnosis of malignancies. New methods must meet the following requirements: reproducibility, low cost, independence from the human factor, as well as the possibility of use in non-specialized clinics by primary care doctors (therapists, otolaryngologists, dentists).

In the last decade, to solve the problems of early cancer diagnosis, researchers have been investigating various non-invasive methods based on the identification and analysis of potential markers that indicate the presence of a malignant neoplasm in a patient’s body. It is known that the development of a malignant neoplasm in the body changes metabolic processes. Metabolites can be cleared from the body in various ways. For instance, volatile and semi-volatile metabolic products are often formed, which are then exhaled. Thus, some specific decay products (metabolites) are present in the exhaled air, and their presence and composition can serve as evidence of a possible malignant process in the body [8,9,10,11,12,13,14,15,16,17,18,19,20]. For example, Philipp Opitz and Olf Herbarth [9] described a highly specific method to discriminate between patients with head and neck tumors and healthy people via the detection of volatile metabolites due to the metabolism of uric acid and urine derivatives. An experimental study was published on the use of volatile organic substances in the exhaled air and in air from the oral cavity as biomarkers to identify patients with squamous cell carcinoma of the oral cavity [10].

Leunis N. and co-authors [11] published a pilot study on the possibility of using an “electronic nose” device in the diagnosis of head and neck tumors. The study showed the significant difference in volatile organic matter composition between patients with diagnosed head and neck cancer and patients in the control group, with 90% sensitivity and 80% specificity. The authors concluded that the use of an “electronic nose” is a potentially promising method in the diagnosis of head and neck cancer due to its speed, simplicity and non-invasive nature [11].

Lung cancer is a complex disease and there may be different histological features, and the lung cancer biomarkers identified by different studies are largely controversial. Thus, M. Phillips [12], one of the pioneers in breathing research, noted that the main biomarkers were usually alkane derivatives. In the study by Song et al. [17] it was found that adenocarcinoma patients have higher concentrations of 1-butanol and 3-hydroxy-2-butanone. Corradi et al. [18] compared the VOC profile between different histological types and showed higher levels of hexane and ethylbenzene in adenocarcinoma compared to squamous cell carcinoma. However, at the same time, in many other studies no association was found between alkane content and lung cancer [15,16]. Hakim et al. considered the possible biochemical pathways of lung cancer development associated with VOCs [19]. There are different reasons for these inconsistencies. Studies vary greatly in terms of breath sampling procedures, study methods (control group selection, patient selection, etc.) and data processing methods.

Creating a standardized practice for methodological issues is a challenge and requires the collective efforts of all researchers in this field. In 2017, the European Respiratory Society published a technical standard for exhaled biomarkers in lung disease [20], and identified several key areas for future research. Finding cancer biomarkers is a complex and long-term process. The resolution of this problem is possible due to the use of a sensor-based gas analyzer in conjunction with neural network data analysis methods.

## 2. Materials and Methods

The purpose of this paper is to study samples of exhaled air obtained from patients with various types and stages of malignancies of the oropharyngeal region, larynx and lungs, as well as to study and search for general signaling markers of diseases that can be detected using an artificial neural network, ensuring the uniformity of the sampling procedure based on a standardized sensor-based gas analysis system [21].

### 2.1. Description of Test Groups

The study was approved by the Bioethical Committee of the Cancer Research Institute, Tomsk National Research Medical Center of the Russian Academy of Sciences (Order on creation No. 57-p dated 23 December 2010). During the study, exhaled air samples were taken from 121 subjects aged from 22 to 95 years. All subjects in the study were divided into two groups: the first test group and the second control group.

Such parameters as age, gender, smoking and history of alcohol consumption, comorbidities, and long-term medication that subjects may be taking for chronic diseases were not considered when forming the test groups and during subsequent analysis of study data. The study and control groups were similar by sex and age.

The test group included patients with morphologically verified malignancy of the oropharyngeal region, larynx or lungs of stage T1-4T0-3M0-1 (52 subjects). The data on samples taken from subjects of the test group are presented in Table 1.

In the group with malignant pathology, male patients prevailed (*n* = 43; 82.7%). The number of female subjects was only 9 (17.3%). The largest number of cases (21 patients) were lung cancer, while the number of patients with oropharyngeal or laryngeal cancer varied from 1 to 9 depending on tumor localization (oropharyngeal, tongue, oral cavity, larynx and mucosa of the lower jaw). The average age in the test group was 60 years.

The control group (Table 2) included subjects who had no clinical data on the presence of malignant pathology at the time of the study (from anamnesis or according to earlier examination data, if any). The non-inclusion criteria for the control group were malignant disease in past medical history, any cancer treatment in past medical history, age under 18 years, acute phase of infectious disease, antibiotic treatment, and pregnancy or breastfeeding.

In the control group, female subjects prevailed (*n* = 55; ~80%), and the number of male subjects was 14 (~20%). Among patients in this group, the average age was 50 years.

### 2.2. Diagnostic Methods to Confirm Diagnosis

All patients underwent a comprehensive examination to clarify the extent and stage of the tumor according to current international standards and recommendations for the diagnosis of malignancies. For this purpose, endoscopic diagnostics (fiber optic bronchoscopy, fiber optic laryngoscopy) and radiological imaging (spiral computed tomography and magnetic resonance imaging) were used, as well as the mandatory morphological verification of tumors by biopsied material.

Radiological imaging was performed on a Siemens Magnetron Essenza 1.5 T magnetic resonance tomograph and Siemens Somatom Emotion 6 computer tomograph (Heusenstamm, Germany). Endoscopic diagnostic methods were implemented using two devices: an OLYMPUS EVIS EXERA II Series 180 endoscopic tower using Olympus bronchoscopes (diameter 4.8 mm) (Tokyo, Japan) and a Karl Storz TELE PACK endoscopic video unit using a rigid tele-laryngoscope (diameter 5.8 mm, angle of view 70°) and a fiber optic rhino-pharyngo-laryngoscope (diameter 3.5 mm) (Tuttlingen, Germany).

Ultrasonic examination of lymph nodes of the neck and abdominal organs was carried out on an Aloka SSD 5500 system using a linear sensor with a frequency of 10 MHz and a convex sensor with a frequency of 3.5 MHz, using polypositional scanning in grayscale (B-mode) and color Doppler mapping (CDM) in real time (Tokyo, Japan).

### 2.3. Developed Method of Analysis and Experimental Diagnosis Method

Prior to the collection of exhaled air samples, patients refrained from eating and drinking, did not use any personal care products (such as perfumed soap or perfume), and refrained from smoking and brushing their teeth at least two hours before the study. The optimal sampling time was during the morning hours. All samples from the control and test groups patients were taken in the morning, immediately after waking up to minimize the impact of physical activity on results

The study was conducted in a specially designated room where the sensor-based gas analysis system was installed and normal environmental conditions were maintained. Each subject signed the informed consent form, and subjects’ baseline data were entered into the database, including age, sex, tumor localization and stage, smoking and alcohol consumption history, comorbidities, and long-term medication that subjects may be taking for chronic diseases [21]. Then the patient in a sitting position exhaled air with maximum exhalations into a special sterile two-layer sample bag with a volume of 5 L. The outer layer was made of ethylene–vinyl alcohol (EVOH) material 90 microns thick and the inner layer was made of very low-density polyethylene (VLDPE) 50 microns thick. The time interval from the moment of sampling to its processing by the device did not exceed 12 h.

A gas analysis complex was developed that is able to analyze gas samples in two modes—direct breathing into the chamber, or the use of bags for gas samples. In this work, we used the remote bag sampling due to the COVID-19 pandemic.

This system consists of a cylindrical sampling chamber (a quartz glass tube which is closed at both ends by brass flanges),with a volume of 1 L, inside which is a module containing 14 MOS sensors, 6 constantly running fans distributing sample air evenly throughout the chamber, and a control board. Controlled inlet and outlet valves are connected to the sampling chamber, as well as a purge system consisting of a compressor and a filter with a mixture of zeolite and silica gel.

The sample bag is connected to the inlet valve. External mechanical pressure affects the bag, and the inlet and outlet valves open for one second. The automation of the collection process and the constant pressure on the sample bag ensured the same volume of air (250 mL) is introduced into the sampling chamber.

After the sample is injected into the chamber, the inlet and outlet valves are closed and the process of collecting data from the sensors with a frequency of 50 Hz for 90 s begins. During the operation of the measuring module, the sensors operate in the thermal cycling mode, and during the data collection, information of thermal cycling is transmitted about 9 times. Upon completion of the data collection process, the device is purged with purified air using a compressor connected to the sampling chamber through a filter [21].

During the study [21], the data obtained by digitizing signals from the sensors of the gas analysis system and patient metadata were recorded in the database for subsequent automated processing and analysis. After the sampling procedure, patients remained under observation for 30 min to assess their overall condition.

For each subject, a separate subdirectory is allocated in the gas analyzer, in which all files containing metadata and arrays of signal data from sensors are stored, thus forming a tree of experimental directories. Communication between the gas analyzer operator software and the database is provided using standard SQL queries. To obtain the result when diagnosing patients with the gas analyzer, signals from 14 sensors were pre-processed and then became available for statistical analysis.

For the purposes of the study, the most suitable type of neural network architecture for classifying patients was a direct distribution neural network (perceptron type). The input layer of the neural network corresponded to the number of pre-processed values of the output signals from the sensors; the output layer corresponded to the number of classification features. In our study, the output layer of the neural network included one neuron taking the value “1” in the presence, and the value “1” in the absence, of malignancy.

**Figure 1:**
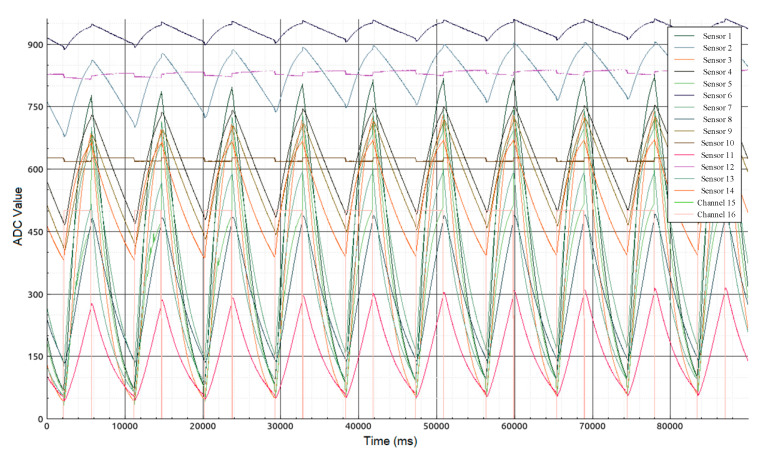
Form of sensor signals (ADC—analog-to-digital converter).

The neural network input was supplied in ratios of 4 and 1 (Figure 1) of the thermal cycling periods of all 14 sensors from the cooling–heating point to the heating–cooling point (3.5 s × 50 Hz = 175 values). In the course of the study it was found empirically that taking each 10th value does not increase the error, thus the size of the input data array was significantly reduced from 175 to 17 values per sensor. Thus, 17 × 14 values obtained from the gas sensors were supplied to the input of the neural network. The total dimension of the input layer of the neural network was 238 values. The dimension of the hidden layer was input twice, resulting in 476 values. Only the values of signals from sensors were supplied to the input of the neural network, without data on the subjects’ sex, age and other factors.

When determining the optimal parameters of neural network training, it was found experimentally that the smallest error on the training and test sets occurs with both a large number of learning epochs and a low learning rate. The best results were obtained with the following settings of the neural network: 1,000,000 epochs of learning with a forced stop when the error on the training set starts decreasing, and the error on the test set starts increasing, to prevent retraining; 0.01 was training rate, and there was 1 hidden layer with dimension 2× input. The activation function for all layers is a hyperbolic tangent. The neural network was trained using the error backpropagation method. The indicator for searching optimal parameters was AUC-ROC (area under ROC curve, where ROC means receiver operating characteristic).

## 3. Results

**Figure 2:**
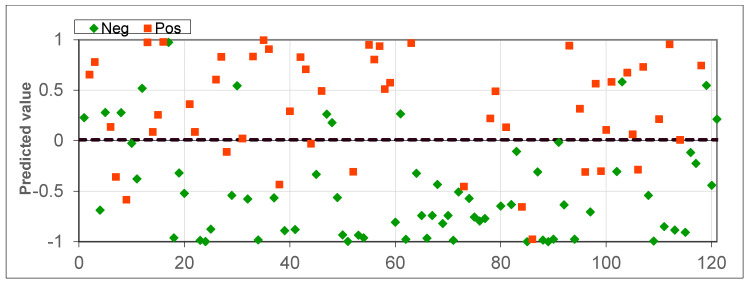
Scatter plot for all healthy patients, patients with laryngeal or oropharyngeal cancer, and patients with lung cancer.

**Figure 3:**
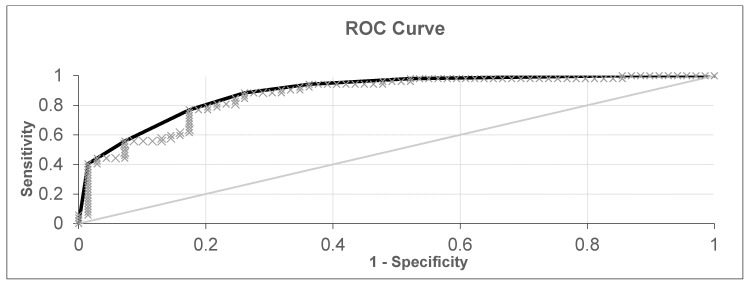
ROC (receiver operating characteristic) curve for all healthy patients, patients with lung cancer, and patients with laryngeal or oropharyngeal cancer (black curve—ROC curve, grey line—Reference line and grey x—experimental points).

The first experiment analyzed a dataset from 69 healthy patients, 31 patients with laryngeal or oropharyngeal cancer, and 21 patients with lung cancer, for a total of 121 samples. After cross-validation, the results shown in Figure 2 and Figure 3 were obtained. The diagnostic accuracy was 80.16%, sensitivity was 76.92%, and specificity was 82.61%.

In the following scatter plots of individual predictive values, the abscissa is the serial number of a patient participating in the cross-validation. The ordinate is the value obtained by the neural network during cross validation. The threshold for separating positive and negative tests was found via analysis of the ROC curve (Figure 3) and was equal to 0.095. If the obtained value was higher than the threshold, then the neural network classified the sample as pathological, but if it was lower than it was classified as without pathology. The type of markers in the figure determines the actual state of the sample; a triangle means without pathology, and a square means with pathology. The extreme values –1 and 1 are provided by the activation function, the hyperbolic tangent, which provides a greater number of intermediate states relative to the sigmoid, where similar values are 0 and 1. All available patient data participated in the cross-validation. The value of AUC-ROC (Figure 3) was 0.867, indicating the high quality of the classifier.

In experiments 2 and 3, on the classification of patients with lung cancer and laryngeal or oropharyngeal cancer, respectively, the pathology/healthy ratio was adjusted to 50/50. Such ratio is necessary due to the predominance of data on healthy patients. With a significant predominance of healthy patients over patients with pathology, the neural network, when trained, will rely on errors obtained in identifying patients with pathology in the test sets. As a result, the accuracy will be low, while the trained classifier will be able to diagnose positive results, i.e., will have a low sensitivity of 30% to 50%. In experiments 2 and 3, healthy patients from the control group were randomly selected from a common dataset.

**Figure 4:**
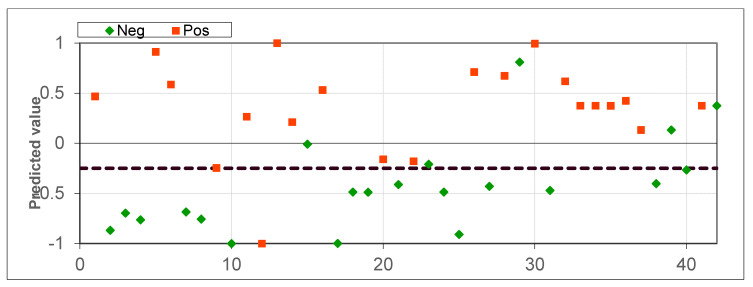
Scatter plot for healthy patients and lung cancer patients.

**Figure 5:**
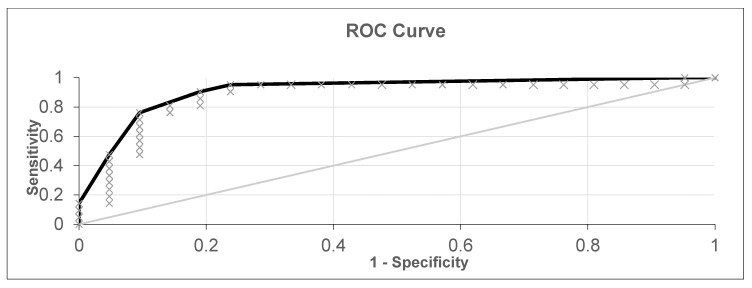
ROC curve for healthy patients and lung cancer patients (black curve—ROC curve, grey line—Reference line and grey x—experimental points).

The cross-validation results for datasets consisting of 21 healthy patients and 21 lung cancer patients (experiment 2) are presented in Figure 4 and Figure 5, totaling 42 samples. The accuracy was 85.71%, the sensitivity was 95.24% (1 patient misclassified) and the specificity was 76.19%. The separation threshold of 0.25 was obtained from the ROC curve. The AUC-ROC was 0.875.

**Figure 6:**
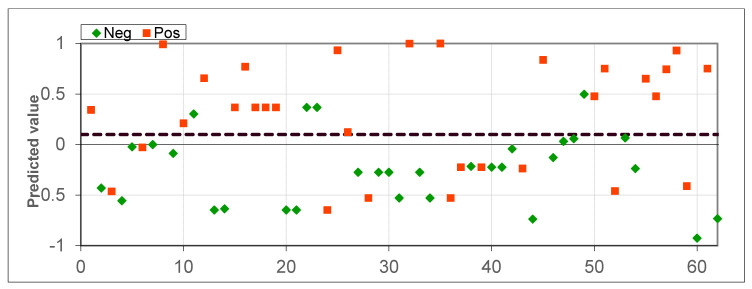
Scatter plot for healthy patients and patients with laryngeal or oropharyngeal cancer.

**Figure 7:**
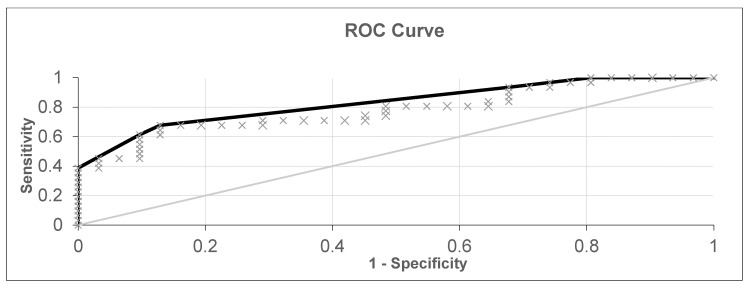
ROC curve for healthy patients and patients with laryngeal or oropharyngeal cancer (black curve—ROC curve, grey line—Reference line and grey x—experimental points).

Experiment 3 was conducted based on data on 31 patients with laryngeal or oropharyngeal cancer and 31 healthy patients, totaling 62 samples. The results are shown in Figure 6 and Figure 7.

The accuracy was 77.41, the sensitivity was 67.74, and the specificity was 87.1. The separation threshold obtained from the ROC curve was 0.1, and the AUC-ROC was 0.783.

From the data obtained, it can be concluded that for the gas analytical complex used and the corresponding study methodology, lung cancer can be determined with the least difficulty.

A summary of the experiments and their results is presented in Table 3. The accuracy, sensitivity and specificity for three different variants of patient datasets were tested using applicable techniques [22,23].

Taking the results into account, the difference in the values of sensitivity and specificity for oropharyngeal cancer and laryngeal cancer may be due to the presence of more informative markers in lung cancer, which are captured by the sensory system and differentiated by the neural network with less error. On the other hand, the somewhat underestimated measures of sensitivity and specificity in our study for tumors of the oropharyngeal region and larynx may be due to the heterogeneity of this group. Tumors of the oral cavity, pharynx and larynx are conventionally united by common localization in the upper respiratory tract, however there are differences in the ontogenetic, therapeutic and prognostic aspects. For example, organs in the oropharyngeal region and larynx develop from different embryonic tissues (germs). Therefore, these organs have different origins and structures, which affect their functional activity, and, therefore, metabolism. Furthermore, tumors of the oropharyngeal region have a number of features, which distinguish them from tumors of the larynx. For example, malignant tumors of the pharynx and oral cavity are prone to frequent regional metastasis (up to 50–70%) compared to malignant tumors of the larynx (metastases to regional nodes are detected in 30–40% of cases). In turn, malignant pharyngeal tumors are most sensitive to chemotherapy and radiation therapy, compared to malignant tumors of the oral cavity and larynx.

## 4. Conclusions

In our study, exhaled air samples were taken from groups of patients with the most common localizations of malignancies (lungs, oropharyngeal region and larynx). At the same time, the largest number of cases (21 patients) were lung cancer, while the number of patients with oropharyngeal or laryngeal cancer varied from 1 to 9 depending on tumor localization (oropharyngeal, tongue, oral cavity, larynx and mucosa of the lower jaw).

In the case of lung cancer, the parameters of the diagnostic device are determined as follows: sensitivity—95.24%, specificity—76.19%. For oropharyngeal cancer and laryngeal cancer, these parameters were 67.74% and 87.1%, respectively. It was shown that study subjects with tumors of the upper respiratory tract (oral cavity, pharynx and larynx) compose a rather heterogeneous group, and this fact can affect the metabolism of tumor tissues and the molecular composition due to the presence of the tumor.

At the current stage of the study, the specificity and sensitivity measures of the method investigated are comparable to those of the existing high-precision radiological methods for diagnosing airway tumors (MRI, SCT, PET). According to the available sources of information, the specificity of PET with 18F-FDG and MRI in the diagnosis of head and neck tumors ranges from 43% to 95%, with sensitivity ranging from 64% to 92% [24]. These values are very similar to those obtained using PET/CT and CT in the diagnosis of lung cancer. Computed tomography has a sensitivity of 72.1% and a specificity of 90.3%. PET/CT has a sensitivity and specificity of 90.1% and 96.2%, respectively [25].

Given the known diagnostic value of such accurate and expensive diagnostic methods as CT and MRI, the sensitivity and specificity of the sensor-based gas analytical system achieved during this study clearly demonstrate the prospects of the proposed technique in the diagnosis of tumor processes in patients with lung cancer, and cancers of the oropharyngeal region and larynx. The distinctive features of the method that we studied include the mobility of equipment used and the possibility of placement in medical institutions of different levels. The method is fast and eliminates the human factor. One of the important advantages of the sensor-based gas analytical system is the simplicity and relative inexpensiveness of the diagnosis, and the possibility of unconstrained use for the purpose of screening for tumor processes in a wide population. The use of this screening method will allow the selection of persons needing a detailed examination using traditional diagnostic methods (endoscopic, radiological and morphological); this will increase the effectiveness and timeliness of detecting malignant neoplasms at an early stage, and allow function-preserving treatment while maintaining a high quality of life.

## Figures and Tables

**Table 1:** Characteristics of the study set of patients.

	*n*	%
Number of patients with malignant pathology	52	43.0
Mean age	60	
Sex		
Male	43	82.7
Female	9	17.3
TNM* staging		
I	8	15.4
II	23	44.2
III	15	28.8
IV	6	11.6
Localization		
Lungs	21	40.4
Larynx	9	17.3
Oral cavity	5	9.6
Oropharynx	7	13.5
Hypopharynx	1	1.9
Tongue	6	11.6
The mucous membrane of the alveolar process of the lower jaw	3	5.7
Tobacco consumption		
Yes	29	55.8
No	23	44.2

TNM* System—TNM Classification of Malignant Tumors.

**Table 2:** Characteristics of the control set of patients.

	*n*	%
Control group (with no data on malignant pathology)	69	57.0
Mean age	50	
Sex		
Male	14	20.3
Female	55	79.7

**Table 3:** Achieved parameters of the gas analysis system.

N	Data Set Parameters	Accuracy	Sensitivity	Specificity
1	69 healthy subjects and 31 subjects with laryngeal or oropharyngeal cancer + 21 subjects with lung cancer	80.16%	76.92%	82.61%
2	21 healthy subjects and 21 subjects with lung cancer	85.71%	95.24%	76.19%
3	31 healthy subjects and 31 laryngeal or oropharyngeal cancer	77.41%	67.74%	87.1%
